# Rapamycin does not inhibit human cytomegalovirus reactivation from dendritic cells *in vitro*

**DOI:** 10.1099/vir.0.066332-0

**Published:** 2014-10

**Authors:** Thomas E. Glover, Verity G. Kew, Matthew B. Reeves

**Affiliations:** 1 Department of Medicine, Addenbrooke’s Hospital, University of Cambridge, Hills Road, Cambridge CB2 0QQ, UK; 2 Institute of Immunity & Transplantation, Royal Free Hospital, University College London, Hampstead, London NW3 2PF, UK

## Abstract

Human cytomegalovirus (HCMV) infection and reactivation are a major cause of morbidity in immune-suppressed patients. Interestingly, epidemiological studies have shown that patients administered the mammalian target of rapamycin (mTOR) inhibitor, sirolimus (rapamycin), exhibit more favourable outcomes, suggestive of activity against HCMV *in vivo*. Given its relative lack of activity against lytic infection, it is postulated that rapamycin inhibits HCMV reactivation. Here, we showed that rapamycin administered acutely or chronically has little impact on induction of immediate early (IE) gene expression in experimentally latent dendritic cells or cells from naturally latent individuals. Furthermore, we extended these observations to include other inhibitors of mTORC1 and mTORC 2, which similarly have minimal effects on induction of IE gene expression from latency. Taken together, these data suggest that favourable outcomes associated with sirolimus are attributable to indirect effects that influence HCMV reactivation, rather than a direct mechanistic action against HCMV itself.

Human cytomegalovirus (HCMV) reactivation is a major cause of disease in transplant recipients and critically ill patients (Legendre & Pascual, 2008; Limaye *et al.*, 2008). Similarly, primary infection poses a major health threat to immunocompromised populations and represents the predominant viral cause of congenital disease, particularly in the developed world (Revello & Gerna, 2004). Thus, understanding the mechanisms underlying HCMV infection and pathogenesis is of significant clinical importance.

HCMV reactivation generally occurs sporadically but at subclinical levels due to the controlling presence of a robust immune response (Jackson *et al.*, 2011; Jost & Altfeld, 2013; Rölle & Olweus, 2009), rendering immune suppression a major factor in clinical reactivation (Smith & Khanna, 2013; Watkins *et al.*, 2012). Although studies systematically correlating HCMV disease incidence with different immune suppression methods have been performed (Chakrabarti *et al.*, 2002, 2004; Lin *et al.*, 2002), the majority of findings require further investigation for unequivocal interpretation. Interestingly, accumulating data have provided evidence of better outcomes upon immune suppression with sirolimus (rapamycin) following both stem cell (Marty *et al.*, 2007) and solid organ transplantation (Demopoulos *et al.*, 2008; Ghassemieh *et al.*, 2013), suggesting that this immunosuppression regimen has a direct impact on HCMV.

Targets of rapamycin (Tor1 and Tor2) were originally identified as yeast proteins sensitive to a naturally occurring antifungal agent expressed by *Streptomyces hygroscopicus* (Heitman *et al.*, 1991; Vézina *et al.*, 1975), and subsequent studies revealed a mammalian target (mTOR) particularly active against the mTOR complex 1 (mTORC1) arm (Heitman *et al.*, 1991; Sabatini *et al.*, 1994). mTOR is a serine/threonine kinase controlling a range of cellular functions, including cell growth, proliferation and survival and affecting transcription and protein synthesis (Lamming *et al*., 2013). Two functional complexes exist: mTORC1, classically described as rapamycin-sensitive and important for stimulation of protein synthesis via activation of p70S6 kinase 1 and 4E-BP1, and a second less well-characterized mTORC2 complex that is generally considered rapamycin-insensitive (Loewith *et al.*, 2002) and implicated in cytoskeletal organization as well as mediation of AKT signalling (Kim *et al.*, 2002; Sarbassov *et al.*, 2005).

Viral targeting of the mTOR pathways during lytic infection positively influences viral replication (Clippinger *et al.*, 2011; Kudchodkar *et al.*, 2004, 2006; Moorman & Shenk, 2010). Interestingly, while HCMV utilizes the mTORC1 pathway, rapamycin has a minimal impact on viral replication in fibroblasts, since HCMV activates a rapamycin-insensitive pathway (Kudchodkar *et al.*, 2004; Moorman & Shenk, 2010). Within 12 h, phosphorylation of 4E-BP1 becomes insensitive to rapamycin, as shown by Kudchodkar *et al.* (2004). Reconciling these data with improved prognosis regarding HCMV infection for patients immunosuppressed with sirolimus led to the proposal that sirolimus abrogates HCMV reactivation and disease *in vivo* by preventing reactivation of HCMV IE gene expression (Marty *et al.*, 2007).

Clearly, for mTOR to be important in HCMV reactivation, it is required to be active in DC (dendritic cell) differentiation. Western blot analysis of mTOR autophosphorylation (anti-phospho-mTOR antibody (Ser2448), 1 : 750, cell signalling) in DCs derived from monocytes (isolated from healthy volunteers under ethical approval from the Cambridge Local Research Ethics committee) suggested that mTOR is active in these cells (Fig. 1a), and enhanced threefold by the addition of LPS (Fig. 1a; densitometry using Image J Software, NIH). Having established mTOR activity in DCs, we examined the effects of rapamycin on reactivation. Latently infected monocytes differentiated into immature DCs (Reeves & Compton, 2011) were treated with log dilutions of rapamycin for 1 h (10 µM–100 nM in DMSO), prior to reactivation. At 24 h post-reactivation, no overt inhibitory effect on immediate early (IE) gene expression was observed using real time qPCR (RT-qPCR) at the lower doses of rapamycin (Fig. 1b), which are higher than those achieved clinically (20–50 nM). However, we noted a trend suggesting that rapamycin has a minor inhibitory effect on IE gene expression following an analysis in cells from multiple donors, although the differences in expression when all four donors were taken together were non-significant (Fig. 1d; *P* = 0.16). Elevated IE gene expression at higher doses of the drug (Fig. 1b) may be linked to the observation that rapamycin at high concentrations mimics or enhances aspects of the inflammatory response, even in the absence of inflammatory cytokines (Barilli *et al.*, 2008; Turnquist *et al.*, 2010) or that activation of death pathways (Fig. 1c) indirectly stimulates HCMV gene expression.

**Fig. 1.**
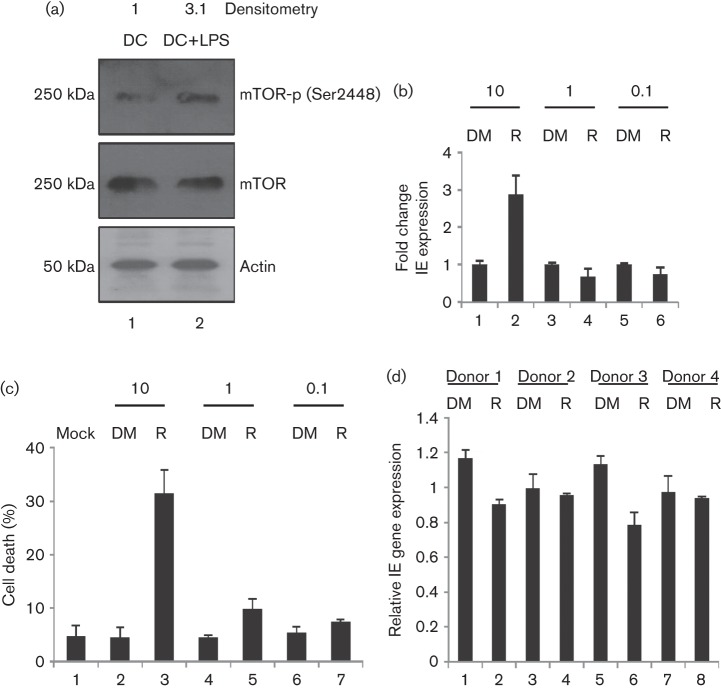
Rapamycin does not inhibit reactivation of HCMV gene expression. (a) Western blot analysis of phosphorylated and total mTOR in immature DCs (1) and mature DCs (2). (b) RNA isolated from immature DCs stimulated with LPS was analysed for IE and GAPDH RNA expression using qRT-PCR. Prior to reactivation, cells were incubated for 2 h with DMSO (1, 3, 5) or rapamycin (2, 4, 6). (c) Immature DCs (1) incubated with DMSO (2, 4, 6) or rapamycin (3, 5, 7) were analysed for viability using trypan blue at 24 h post-treatment. (d) RNA isolated from MoDCs derived from four independent monocyte donors was analysed for reactivation of experimental latency via qRT-PCR post-LPS stimulation in the presence of 1 µM rapamycin (2, 4, 6, 8) or DMSO control (1, 3, 5, 7). IE gene expression was determined, relative to untreated (no solvent) LPS control. Statistical analysis was performed on the four donors combined (*n* = 4; *t*-test, *P = *0.16).

Pre-treatment of fibroblasts with another mTORC1 inhibitor, AICAR (aminoimidazole carboxamide ribonucleotide), has been shown to block MIEP (major IE promoter) activity, but only if added prior to HCMV infection (Kudchodkar *et al.*, 2007). Furthermore, AICAR is deleterious to viral replication during lytic infection, suggesting that modulation of the cellular AMP : ATP level affects HCMV infection (Kudchodkar *et al.*, 2007). AICAR acts as an agonist of AMP-activated protein kinase (AMPK) through modulation of cellular AMP : ATP levels (Corton *et al.*, 1995). AICAR couples elevated AMP levels (and thus cellular energy deprivation) with increased AMPK phosphorylation and activation (Luo *et al.*, 2005), resulting in inactivation of mTOR pathways. Accordingly, we examined whether pre-treatment of immature DCs with AICAR affects their response to reactivation stimuli (Fig. 2). Immature DCs pre-treated with rapamycin (1 µM) or AICAR (0.5 mM) at non-toxic concentrations (Figs 1c and 2b) that suppress mTOR activation (Fig. 2a) were stimulated with LPS or IL-6, and reactivation of IE gene expression measured using RT-qPCR. Again, no appreciable effect of rapamycin on HCMV reactivation was observed following IL-6 or LPS stimulation whereas a minor effect was evident with AICAR (Fig. 2c). To assess infectious virus production after reactivation, DCs were cultured on a monolayer of human foreskin fibroblasts (HFFs) at 5 days post-reactivation for a further 5 days. Co-culture supernatants were tested for the presence of infectious virus by inoculation with fresh indicator fibroblasts and subsequent scoring of IE positivity. Consistent with IE RNA expression data, no significant impact on virus production was observed in DCs cultured with rapamycin (Fig. 2d; *P*>0.05). However, a statistically significant (twofold to threefold) decrease in virus production was detected in AICAR-treated reactivated DCs (Fig. 2d; *P*<0.05). Interestingly, earlier studies on HFFs have identified a post-IE effect of AICAR on virus production during lytic infection (Kudchodkar *et al.*, 2007). Finally, these inhibitors appeared to have no significant impact on induction of IE gene expression from latent HCMV in cells of seropositive donors (Fig. 2e), suggesting that our observations with experimental latency can be replicated in natural latency using previously defined protocols (Reeves & Compton, 2011).

**Fig. 2.**
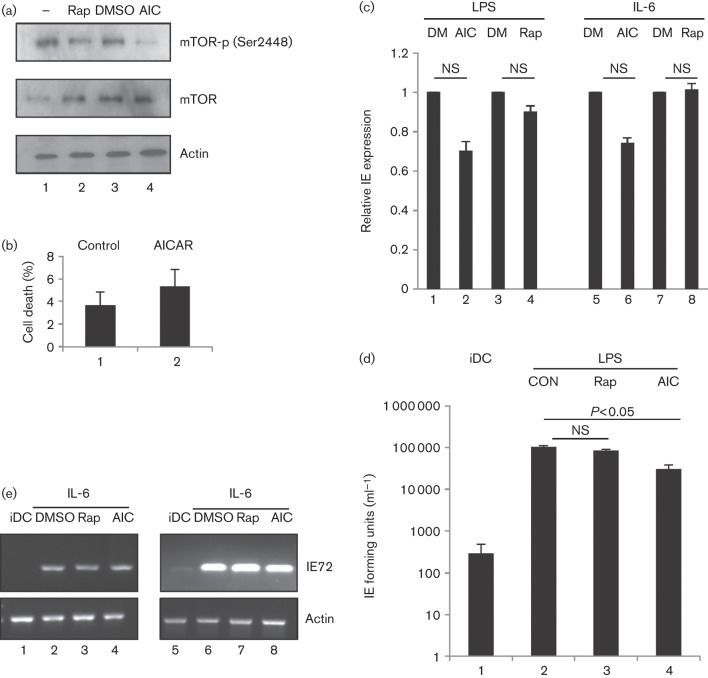
Inhibition of mTORC1 using AICAR promotes a minor defect in virus reactivation. (a) Western blot analysis of mTOR phosphorylation in DCs (1) or DCs incubated with 1 µM rapamycin (2), DMSO (3) or 0.5 mM AICAR (4) for 3 h. (b) Immature DCs incubated with DMSO (1) or AICAR (2) were analysed for viability using trypan blue at 24 h post-treatment. (c) qRT-PCR analysis of RNA isolated from immature DCs pre-treated with DMSO (1, 3, 5, 7; DM), AICAR (2, 6; AIC) or rapamycin (4, 8; Rap) for 2 h, prior to LPS (1–4) or IL-6 (5–8) stimulation. (d) Supernatants from DC : HFF co-cultures at 10 days post-reactivation were used to inoculate fresh fibroblasts and scored for IE positivity as a measure of infectious virus reactivation. Quantification is shown for immature DC (iDC), immature DC+LPS (CON), immature DC+LPS+rapamycin (Rap) and immature DC+LPS+AICAR (AIC). (e) Monocytes isolated from two seropositive donors were differentiated into iDCs (1, 5), and prior to IL-6- induced reactivation (2–4; 6–8), pretreated with DMSO (2, 6), 1 µM rapamycin (3, 7) or AICAR (4, 8). RNA was analysed with nested PCR for IE72 gene expression as described previously (Reeves & Compton, 2011). NS, Non-significant by *t*-test (*n* = 3).

Whilst mTORC1 does not appear to play a major role in reactivation, a role for mTORC2 cannot be discounted. Indeed, studies on an NTera2/D1 quiescent infection model suggest that CREB (cAMP response element binding protein) and mTORC2 interactions promote HCMV MIEP gene expression in response to cAMP activators (Yuan *et al.*, 2009). Furthermore, these studies in cell culture reflect acute exposure to rapamycin due to the nature of the drug administration whereas *in vivo* the exposure is more chronic due to long term use. Importantly, chronic exposure of cells to rapamycin is deleterious to the mTORC2 pathway (Lamming *et al.*, 2012; Sarbassov *et al.*, 2006; Vollenbröker *et al.*, 2009) via blockade of the formation of *de novo* complexes following their natural turnover in the cell (Sarbassov *et al.*, 2006). Accordingly, we addressed whether mTORC2 plays a role in reactivation.

OSI-027 (Selleckchem; 10 µM in DMSO) is a well-characterized inhibitor of mTOR under trial for treatment of leukaemia due to effects on both mTORC1 and 2 *in vivo* (Bhagwat *et al.*, 2011). Immature DCs derived from latently infected monocytes were treated with OSI-027 for 2 h, prior to the induction of reactivation (Fig. 3), at a concentration known to block downstream AKT activation (Fig. 3a) but not trigger cell death (Fig. 3b). However, no significant effects on RNA and protein expression levels were observed (Fig. 3c,d; *P*>0.05, respectively). Similarly, chronic exposure to rapamycin was ineffective at inhibiting HCMV reactivation (Fig. 3e; *P*>0.05), with chronic administration modelled by treating with rapamycin for the final 3 days of the 6-day differentiation period to immature DCs. The data collectively suggest that rapamycin and mTOR signalling have minor involvement in the induction of IE gene expression from DCs *in vitro*.

**Fig. 3.**
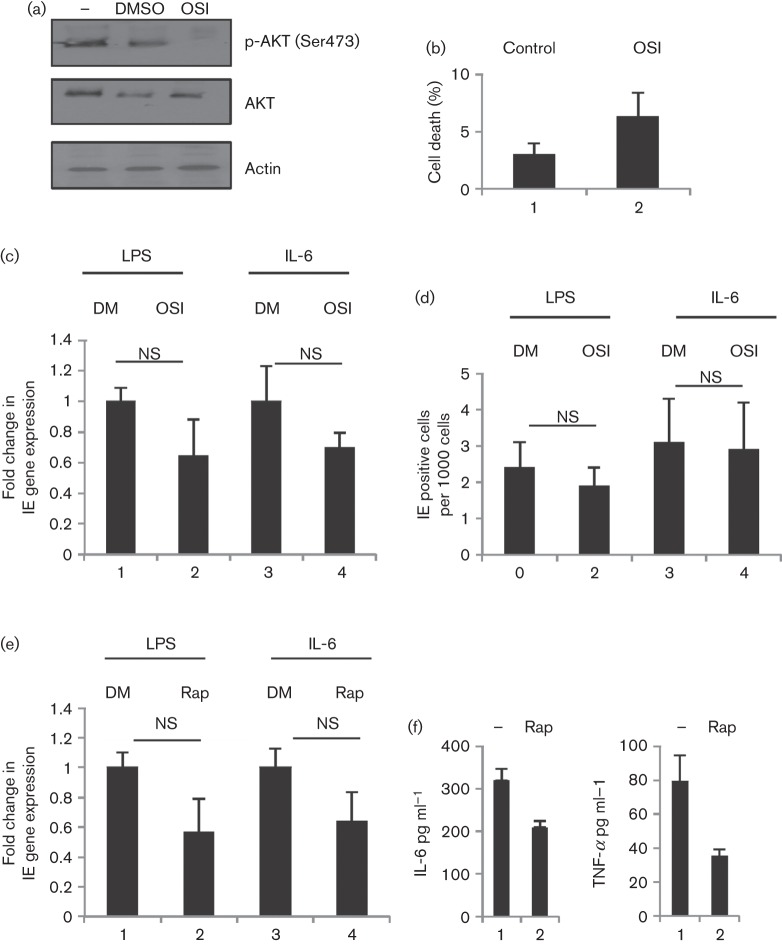
Inhibition of mTORC1 and mTORC2 does not block induction of IE gene expression (a) Western blot analysis for AKT phosphorylation in iDCs (1), iDCs plus DMSO (2) and iDCs plus OSI-027 (3). (b) Immature DCs incubated with DMSO (1) or OSI-027 (2) were analysed for viability using trypan blue at 24 h post-treatment. (c, d) qRT-PCR (c) and immunofluorescence analyses (d) of IE expression were performed on immature DCs pre-incubated with DMSO (1, 3; DM) or OSI-027 (2,4; OSI) for 2 h prior to LPS or IL-6 stimulation. (e) qRT-PCR analysis was performed on RNA isolated from immature DCs pre-incubated with DMSO (1, 3; DM) or rapamycin (2, 4; Rap) for 3 days, prior to LPS or IL-6 stimulation. (f) Immature DCs were incubated with 1 µM rapamycin (2, 4), prior to LPS stimulation and IL-6 (1, 2) and TNF-α expression measured using cytokine ELISA. NS, Non-significant with *t*-test (*n* = 3).

HCMV becomes insensitive to rapamycin within 12 h post-infection (Kudchodkar *et al.*, 2004, 2006; Moorman & Shenk, 2010) and uses a rapamycin-insensitive pathway to maintain viral progression through its life cycle (Clippinger *et al.*, 2011; Moorman & Shenk, 2010). However, many of these earlier studies were performed in fibroblasts (Alwine, 2008), a cell type more resistant to metabolic stress *in vitro* (Baines *et al.*, 2005; Li *et al.*, 2005). This was exemplified by studies on the role of the non-coding RNA beta 2.7 and its effects on cell viability and energy production (Reeves *et al.*, 2007), where a growth defect was observed in neuronal but not fibroblast cells (McSharry *et al.*, 2003; Reeves *et al.*, 2007). Consistent with differential sensitivity, polarized M2 macrophages infected with HCMV were shown to be sensitive to rapamycin, with impaired virus production (Poglitsch *et al.*, 2012). However, the studies presented here suggest that HCMV in DCs are similarly resistant to rapamycin during reactivation.

These observations collectively suggest that better outcomes regarding post-transplant HCMV disease associated with sirolimus-based immune suppression therapies are largely attributed to indirect effects. HCMV reactivation and disease in a clinical setting are possibly dependent on a number of factors. Firstly, there is the rapidity of reconstitution of the immune response (Smith & Khanna, 2013; Watkins *et al.*, 2012), illustrated by the benefits associated with transplanting seropositive donor CD34+ cells to seropositive recipients in HLA-mismatched bone marrow transplants due to the transfer of pre-existing immunity (Ljungman *et al.*, 2003). Secondly, this could affect the resolution of secondary infections potentially exacerbated by HCMV replication (Nichols *et al.*, 2002). Inherent in these observations is the view that the nature of the reconstituted immune response dictates the ability of the host to control viral reactivation (Roux *et al.*, 2000). Clearly, if certain immune suppressive regimens induce accelerated recovery of specific T-cell responses important for controlling HCMV, a clinical advantage exists. Thirdly, several lines of evidence, both *in vitro* and *in vivo*, suggest that inflammation exacerbates HCMV reactivation (Blankenberg *et al.*, 2001; Hargett & Shenk, 2010; Humar *et al.*, 1999; Prösch *et al.*, 2002; Reeves & Compton, 2011; Söderberg-Nauclér *et al.*, 1997). A major source of inflammation is from allogeneically stimulated T cells, an event shown to promote HCMV reactivation *ex vivo* (Söderberg-Nauclér *et al.*, 1997). Indeed, reduced incidence of graft versus host disease (GvHD) observed with sirolimus (Antin *et al.*, 2003; Armand *et al.*, 2008) is linked with a high proportion of T regulatory cells produced in these patients (San Segundo *et al.*, 2010). Thus, suppression of CD4+ allogeneic responses protects against GvHD (Hester *et al.*, 2012). Clearly, in the context of HCMV, the provision of a less inflammatory environment may also affect reactivation. Consistently, in our *in vitro* model, the trend towards a minor decrease in IE gene expression upon stimulation with LPS may be linked with reduced inflammation in the presence of rapamycin (Fig. 3f), as measured using IL-6 and TNF-α cytokine ELISA (R&D systems, Abingdon, UK). Finally, a number of immunosuppressive regimens promote increased inflammatory responses. For example, antithymocyte G has been shown to induce inflammatory gene expression from monocytes (Rameshwar & Gascón, 1992). Among the cytokines produced in such a response, IL-6 is linked with HCMV reactivation *in vitro* (Hargett & Shenk, 2010; Huang *et al.*, 2012; Reeves & Compton, 2011) and IL-1b has been shown to trigger MCMV (mouse cytomegalovirus) reactivation (Cook *et al.*, 2006).

In summary, while these data are essentially negative, they address an important theory regarding the improved outcomes associated with sirolimus-based immune suppression. The findings imply that improved outcomes during transplant (Demopoulos *et al.*, 2008; Ghassemieh *et al.*, 2013; Marty *et al.*, 2007) are associated with indirect effects on the immune system, rather than direct molecular blockade of HCMV reactivation *in vivo*.
